# Flexible ureteroscopy in renal anomalies: an explainable AI model for surgical outcome prediction from EAU endourology

**DOI:** 10.1007/s00345-025-05904-x

**Published:** 2025-09-02

**Authors:** Carlotta Nedbal, Vineet Gauhar, Sairam Adithya, Nithesh Naik, Shilpa Gite, Het Sevalia, Daniele Castellani, Andrea Gregori, Frédéric Panthier, Yiloren Tanidir, Anil Shrestha, Vikram Sridharan, Abhishek Singh, Boyke Soebhali, Mohamed Amine Lakmichi, Saeed Biin Hamri, Bhaskar Kumar Somani

**Affiliations:** 1 Present Address: Polytechnic University Le Marche, Ancona, Italy; 2 Endourology Section, European Association of Urology, Arnhem, The Netherlands; 3 Urology, Ng Teng Fong General Hospital, Singapore, Singapore; 4 Engineering, Symbiosis Institute of Technology, Pune, India; 5 Engineering, Manipal Academy of Higher Education, Manipal, India; 6 Azienda Ospedaliero-Universitaria Ospedali Riuniti di Ancona, Ancona, Italy; 7 IRCSS San Gerardo, Monza, Italy; 8 GRC Urolithiasis No. 20, Tenon Hospital, Sorbonne University, Paris, France; 9 PIMM, UMR 8006 CNRS-Arts et Métiers ParisTech, Paris, France; 10 Department of Urology, Marmara University School of Medicine, Istanbul, Turkey; 11 Department of Urology, Bir Hospital, National Academy of Medical Sciences, Kanti Path, Kathmandu, Nepal; 12 Department of Urology, Sree Paduka Speciality Hospital, Thillai Nagar, Tiruchirappalli, India; 13 Muljibhai Patel Urological Hospital, Nadiad, Gujarat India; 14 Department of Urology, Abdul Wahab Sjahranie Hospital Medical Faculty, Muliawarman University, Samarinda, Indonesia; 15 Department of Urology, School of Medicine, Hospital das Clínicas, Federal University of Paraná, Curitiba, Brazil; 16 King Abdullah International Medical Research Centre, Department of Surgery, Riyadh, Saudi Arabia; 17 University Hospital Southampton NHS Foundation Trust, Southampton, UK

**Keywords:** Ureteroscopy, Renal anomalies, Machine learning, Predictive model, Stone free rate, Explainable AI

## Abstract

**Aim and objective:**

Flexible ureteroscopy (fURS) is a well-established modality for managing urolithiasis in patients with congenital renal anomalies such as horseshoe kidneys (HK), malrotated kidneys (MK), and pelvic ectopic kidneys (PEK). Still, these anatomical variants present unique challenges that complicate stone clearance and procedural planning. We aim to apply machine learning (ML) and explainable artificial intelligence (XAI) techniques to identify predictors of stone-free status (SFS) following fURS in patients with anomalous kidneys.

**Methods:**

We retrospectively analysed adult patients with HK, MK, or EK who underwent fURS and laser lithotripsy for renal stones at a tertiary referral center. A ML model incorporating clinical and intraoperative variables was developed to predict SFS. SHAP (SHapley Additive exPlanations) values and decision tree analysis were used to interpret feature importance and model behaviour.

**Results:**

A total of 569 cases were analysed between 2017 and 2021, with a female: male ratio of 3:1. Regarding anatomical anomalies, 50.62% had HSK, 22.67% had PEK and 26.71% had MK. Most of the patients presented with multiple (59.58%), small (76.80%) and soft stones (56.94%). MK showed the highest SFS rates, suggesting this is the most favourable anomaly for fURS. The presence of residual fragments at the end of the procedure was the strongest negative predictor of SFS, followed by longer operative time and older patient age. PEK exhibited the greatest heterogeneity in outcomes. SHAP analysis provided individualized and global insights into feature contributions.

**Conclusion:**

Explainable AI offers a transparent and clinically meaningful approach to predicting SFS in patients with renal anomalies undergoing fURS. These insights can guide preoperative risk stratification and inform surgical strategy in a domain where standardised evidence is lacking.

## Introduction

Flexible ureteroscopy (fURS) has become a widely adopted technique for the management of renal and ureteric calculi [1]. However, its application in patients with congenital renal anomalies, such as malrotated, ectopic, or horseshoe kidneys remains a clinical challenge. Anatomical variations complicate access, increase the risk of complications, and may impact the success of stone clearance [2].

Despite its growing use, and new literature evidence on safety and efficacy of fURSL in congenital renal anomalies, there is a lack of standardized guidelines addressing URS outcomes in these complex anatomical settings, making clinical decision-making highly dependent on individual experience and intraoperative judgment [3]. As reported by the European Association of Urology (EAU) guidelines, all standard treatment lines are feasible, including fURS, percutaneous nephrolithotomy (PCNL) and shock wave lithotripsy [4]. Nevertheless, typical outcomes (especially for horseshoe kidney disease) are usually lower compared to the control population, with acceptable stone free rates (SFRs) (up to 76%). URS is still considered the safest option, with low major complication rates (2.4%) [5].

In this context, predictive modelling using artificial intelligence (AI) and machine learning (ML) algorithms holds promise for improving patient outcomes, stratification and procedural planning. Traditional AI models often function as “black boxes,” providing limited insight into how predictions are generated—an issue that is especially problematic in high-stakes clinical settings [6]. Explainable AI (XAI) addresses this challenge by making model logic interpretable and clarifying the contribution of individual features to the output [7].

This report explores the application of decision trees and SHAP (SHapley Additive exPlanations) values in a model designed to predict outcome of fURS for urolithiasis in patients with known anatomical kidney anomalies. The objective is to highlight the most influential predictors, examine class distribution patterns, and provide clinicians with data-driven insights to support management strategies in anatomically complex scenarios.

## Materials and methods

### Database: patients selection, data collection and procedures

Patient data were retrospectively analysed from individuals who underwent flexible fURS for urolithiasis between 2017 and 2021 in nineteen high volume centers. Operative results were collected in a large multicentric database, and have been presented in a previous study [1]. The study focused exclusively on adult patients diagnosed with congenital renal anomalies, including horseshoe kidney (HK), malrotated kidney (MK), and pelvic ectopic kidney (PEK). Inclusion criteria required patients to have one or more renal calculi and to have been treated with fURS combined with laser lithotripsy (fURSL). Only cases with confirmed anatomical abnormalities and complete procedural records were included in the final dataset.

Preoperative and demographic characteristics were collected, including age, gender, comorbidities, stone number (single or multiple), size (< 20 mm, ≥ 20 mm) and density (< 1000 HU, ≥ 1000 HU). Preoperative imaging consisted of plain or contrast-enhanced CT scan to evaluate anatomy and stone features. Patients undergoing fURS for other diagnosis than urolithiasis (i.e., upper tract tumour), paediatric patients, patients without anomalous kidneys anatomy, and patients not giving consent for data collection were excluded from the study. Every patient received specialistic counselling before surgery, an informed consent and permission towards data registration were routinely obtained. All patients were tested for urine analysis to rule out any ongoing infection, and a sterile sample was required to proceed with surgery. Intraoperative recorded data included: operative time, intraoperative haematuria limiting vision, failed procedure with abandoned operation, presence of on-table residual fragments (RF). After the procedure, the following parameters were collected: postoperative sepsis, need for reintervention, SFR at 3-months follow-up CT-KUB (defined as absence of RF, or RF < 2 mm).

### ML algorithms training and data analysis

The workflow commenced with data cleaning and preprocessing, which included eliminating irrelevant characters and imputing missing categorical values using the mode. Subsequently, statistical analyses were conducted, and these encompassed correlation assessments, Variance Inflation Factor (ViF) evaluations, and logistic regression across four distinct prediction tasks. Next, a suite of eighteen ML models was trained individually. These included Probabilistic Models (Naive Bayes, Logistic Regression), Instance-Based (Lazy) Learning (K-Nearest Neighbors – KNN), Linear Models (Logistic Regression, Linear Discriminant Analysis – LDA, SVM – Linear), Discriminant Analysis (Linear Discriminant Analysis – LDA, Quadratic Discriminant Analysis – QDA), Support Vector Machines (SVM: SVM – Linear SVM – Poly, SVM – RBF), Tree-Based Models (Decision Tree, Random Forest, Extra Trees Classifier, Bagging Classifier, Gradient Boosting, CatBoost Classifier, XGBoost). Each model was designed to predict clinical outcomes based on preoperative features.

In parallel, a multitask artificial neural network (ANN) was constructed to predict all outcomes concurrently. This ANN incorporated shared hidden layers for common feature extraction, along with task-specific output layers, employing ReLU activation functions for hidden layers and sigmoid functions for the outputs. Model evaluation was carried out using confusion matrices and classification reports, yielding key performance metrics such as accuracy, precision, recall, and F1 score.

To enhance the interpretability of complex models like the ANN, explainable AI techniques were applied, specifically SHAP value analysis and feature importance mapping, to clarify the model’s decision-making process and spotlight the most influential input variables.

## Results

### Results from the database

A total of 569 cases were recorded and deemed eligible for the analysis during the observation period. With a mean age of 44.51 years, 73.46% were female and 26.54% male. 122 patients had hypertension (HTN), 125 had diabetes mellitus (DM), and 47 had known cardiovascular disease. Regarding anatomical anomalies, patients with HSK were the majority, accounting for 50.62% of the total, while PEK was present in 22.67% and MK in 26.71% cases. Most of the patients presented with multiple (59.58%), small (76.80%) and soft stones (56.94%). Table 1 summarises preoperative findings.

**Table 1:** Intraoperative findings

	No. (%)	Mean age, years	HTN (*n*, %)	DM (*n*, %)	CVD (*n*, %)	HSK (*n*, %)	PEK (*n*, %)	MK (*n*, %)	Multiple stones (*n*, %)	Large stone(s) (*n*, %)	Hard stone(s) (*n*, %)
Female	418 (73.46%)	44.46	94 (22.49%)	89 (21.29%)	34 (8.13%)	221 (52.87%)	86 (20.57%)	111 (26.56%)	252 (60.29%)	92 (22.00%)	176 (42.11%)
Male	151 (26.54%)	44.66	28 (18.54%)	36 (23.84%)	13 (8.61%)	67 (44.37%)	43 (28.48%)	41 (27.15%)	87 (57.62%)	40 (26.49%)	69 (45.70%)
Total	569	44.51	122 (21.44%)	125 (21.97%)	47 (8.26%)	288 (50.62%)	129 (22.67%)	152 (26.71%)	339 (59.58%)	132 (23.20%)	245 (43.06%)

Overall, 34 patients developed significant haematuria, and operation was abandoned due to impossibility to proceed in 16 cases. 53 patients developed postoperative sepsis requiring prolonged intravenous antibiotics. On-table evaluation deemed patients stone-free in 78.56% cases, with confirmatory CT-KUB at 3-months follow-up revealing SFR at 72.06%. Table 2 shows intraoperative and postoperative results

**Table 2:** Postoperative results divided per type of anatomical anomaly

	Haematuria (*n*, %)	Sepsis (*n*, %)	Abandoned operation (*n*, %)	on-table RF (*n*, %)	Reintervention (*n*, %)	SF (*n*, %)
HSK	18 (6.25%)	27 (9.38%)	10 (3.47%)	70 (24.31%)	30 (10.42%)	214 (74.31%)
PEK	9 (6.98%)	13 (10.08%)	2 (1.55%)	28 (21.71%)	14 ( 10.85%)	77 (59.69%)
MK	7 (4.61%)	13 (8.55%)	4 (2.63%)	24 (15.79%)	13 (8.55%)	119 (78.29%)
Total	34 (5.98%)	53 (9.31%)	16 (2.81%)	122 (21.44%)	57 (10.02%)	410 (72.06%)

### On-table RF

#### ML training and testing results

Gradient Boosting is the top model with 89.47% training accuracy, 73.68% validation accuracy, and the highest F1-score (63.60%), showing strong generalization. Decision Tree, Random Forest, and Extra Trees perform well but may overfit (96.53% training, ~ 72.81% validation). XGBoost (95.84% training, 71.05% validation, 60.46% F1) and CatBoost (91.41% training, 70.17% validation, 58.75% F1) offer balanced performance. Bagging Classifier (95.43% training, 71.05% validation, 59.49% F1) is another good ensemble option. Logistic Regression (75.48% training, 64.04% validation, 59.27% F1) and LDA (76.18% training, 64.92% validation, 58.77% F1) provide consistent performance. AdaBoost (79.36% training, 57.23% validation, 57.35% F1) has a solid F1-score but lower validation accuracy. Gradient Boosting resulted to be the top choice, followed by XGBoost and CatBoost for balanced performance. Logistic Regression and LDA appeared reliable for generalization, while SVM-RBF and Naïve Bayes were not suitable.

#### Analysis of correlation and logistic regression

Reintervention was the strongest predictor of on-table RF (*r* = 0.4686), indicating a high likelihood of RF in cases requiring a second procedure. Other associated factors included longer operative times (*r* = 0.2056), abandoned operations (*r* = 0.2105), and haematuria (*r* = 0.1718). Sepsis (*r* = 0.1254) and HSK (*r* = 0.0752) were also weakly correlated with RF. In contrast, a 3-month complete SFS (*r*=-0.6255) was strongly negatively correlated with on-table RF. Additionally, multiple stones (*r*=-0.1440), larger stone diameter (*r*=-0.0501), and higher HU values (*r*=-0.0071) showed weak negative associations with RF.

The model performance metrics show a Log-Likelihood of -166.66, indicating the model fit, with a higher (less negative) value being preferred. The Pseudo R-squared of 0.1163 suggests that the model explains 11.63% of the variance, which is relatively low. The LLR p-value is highly significant (< 0.001), confirming that the overall model is statistically significant. Additionally, the model successfully converged, with no numerical stability issues.

### Need for reintervention

#### ML training and testing results

CatBoost lead with the highest validation accuracy (91.22%) and strong F1-score (61.94%). AdaBoost follows closely with 90.35% validation accuracy and 60.75% F1, showing minimal overfitting. Gradient Boosting had the highest validation accuracy (92.10%) but a lower F1-score (47.95%), suggesting class imbalance. XGBoost (87.19% validation, 57.77% F1) and Bagging Classifier (87.19% validation, 57.77% F1) performed well but show slight overfitting. Logistic Regression (80.70% validation, 57.78% F1) and LDA (84.21% validation, 56.67% F1) provided decent generalization. KNN (78.07% validation, 50.61% F1) performed reasonably but with a weaker F1-score. CatBoost, AdaBoost, and Gradient Boosting are the top models. XGBoost and Bagging Classifier showed good performance with slight overfitting, while Decision Trees, Random Forest, and Extra Trees show overfitting. SVM-Poly and Naïve Bayes underperformed.

#### Analysis of correlation and logistic regression

On-table RF was the strongest predictor of reintervention (*r* = 0.4066), indicating that patients who demonstrate incomplete clearance during surgery are more likely to require additional procedures. Other moderate risk factors for reintervention include haematuria (*r* = 0.2120), sepsis (*r* = 0.1723), and abandoned operations (*r* = 0.1898). Higher stone density also showed a weak positive correlation (*r* = 0.0523) with reintervention, as did cardiovascular disease (*r* = 0.1067). Conversely, achieving a complete stone free status (SFS) (*r*=-0.2877) significantly reduced the likelihood of needing reintervention. Longer surgeries (*r*=-0.0113) and multiple stones (*r*=-0.0845) showed weak negative correlations, with little impact on the need for additional procedures.

The model performance metrics show a Log-Likelihood of -135.42, indicating the model’s fit, with a higher (less negative) value preferred. The Pseudo R-squared of 0.07306 suggests that the model explains only 7.31% of the variance, indicating relatively low predictive power. The LLR p-value of 0.01880 is statistically significant (*p* < 0.05), confirming that the overall model is significant. Additionally, the model successfully converged without any numerical stability issues.

### Stone free status (SFS)

#### ML training and testing results

LDA was the top performer with the highest F1-score (61.97%) and strong balance between precision and recall. QDA had slightly higher validation accuracy (70.18%) but a lower F1-score (41.23%), indicating class imbalance. Random Forest and Extra Trees showed signs of overfitting despite decent F1-scores (~ 57%). Gradient Boosting (63.16% validation, 55.21% F1) and Bagging (52.44% F1) offered good generalization. Logistic Regression (64.91% validation, 60.52% F1) showed solid, balanced performance. KNN performed moderately (55.92% F1) but with lower validation accuracy. Overall, LDA is the best choice, with Logistic Regression, Gradient Boosting, and QDA also performing well.

#### Analysis of correlation and logistic regression

Larger stone diameter (= 0.6306) and MK (*r* = 0.8384) were the strongest positive predictors of SFS, suggesting that larger stones and certain anatomical anomalies are more likely to result in complete stone removal. A single stone (*r* = 0.0899) also showed a slight positive correlation with SFS. On the other hand, on-table RF (*r*=-0.6255), need for reintervention (*r*=-0.2877), and longer operative times (*r*=-0.1118) were strongly and moderately negatively correlated with SFS, indicating that these factors hinder complete stone removal. Additionally, complications such as haematuria (*r*=-0.0410) and sepsis (*r*=-0.0501), as well as abandoned operations (*r*=-0.0888), showed weak negative correlations, slightly reducing the likelihood of achieving SFS.

The model performance metrics show a Log-Likelihood of -203.83, indicating the model’s fit, with a higher (less negative) value preferred. The Pseudo R-squared of 0.07604 suggests that the model explains only 7.60% of the variance, indicating relatively low predictive power. The LLR p-value of 0.0002200 is highly significant, confirming that the overall model is statistically significant. Additionally, the model successfully converged without any numerical stability issues.

#### XAI

**Fig. 1:**
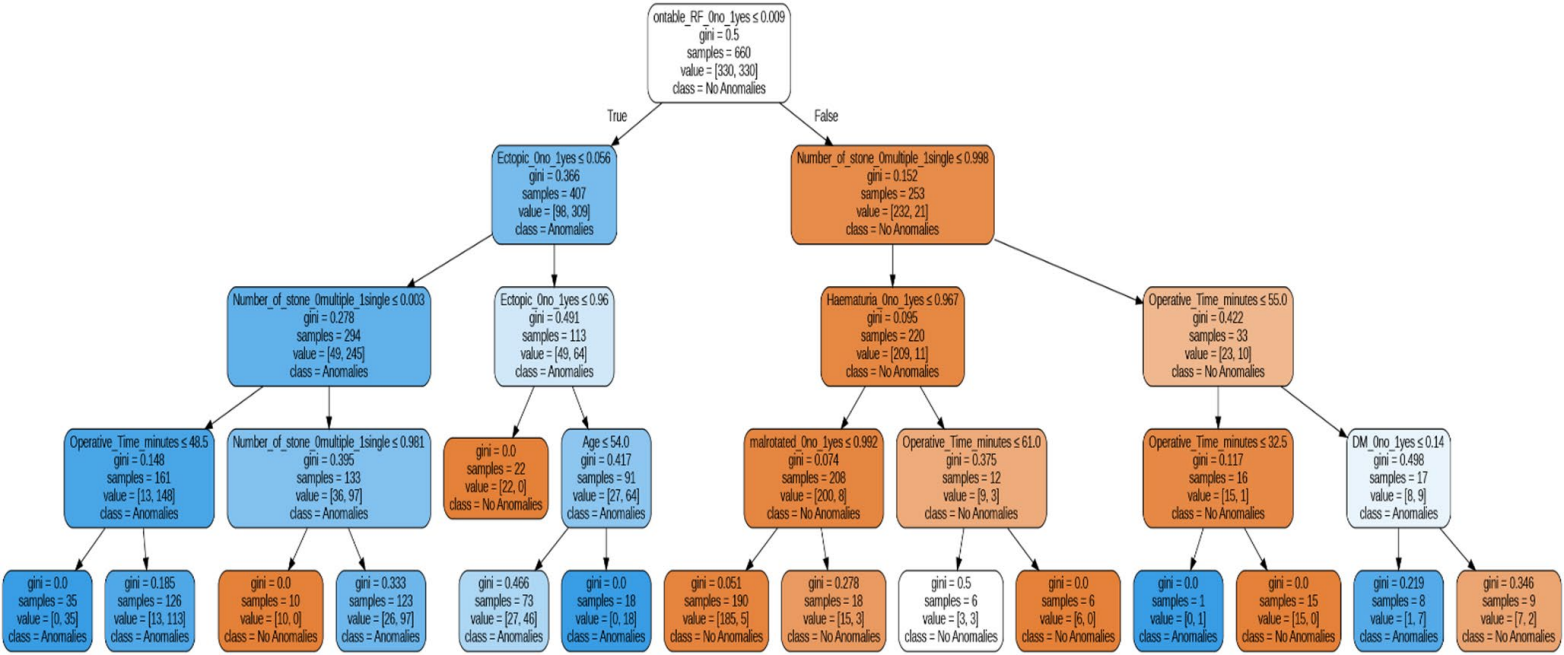
XAI: Decision tree for SFS

In the decision tree model (Fig. 1), several key features were identified as significant predictors of SFS during ureteroscopy. The most important feature, on-table RF, contributed approximately 30% to the model’s prediction. Other notable features included operative time and age, each contributing over 10%. Anatomical factors such as MK were strongly associated with a higher likelihood of achieving SFS, while features like the number of uroliths and the presence of PEK played a crucial role in distinguishing between stone-free and non-stone-free cases. The decision tree model provided a clear classification structure, with decision nodes based on these critical features influencing the likelihood of achieving SFS.

**Fig. 2:**
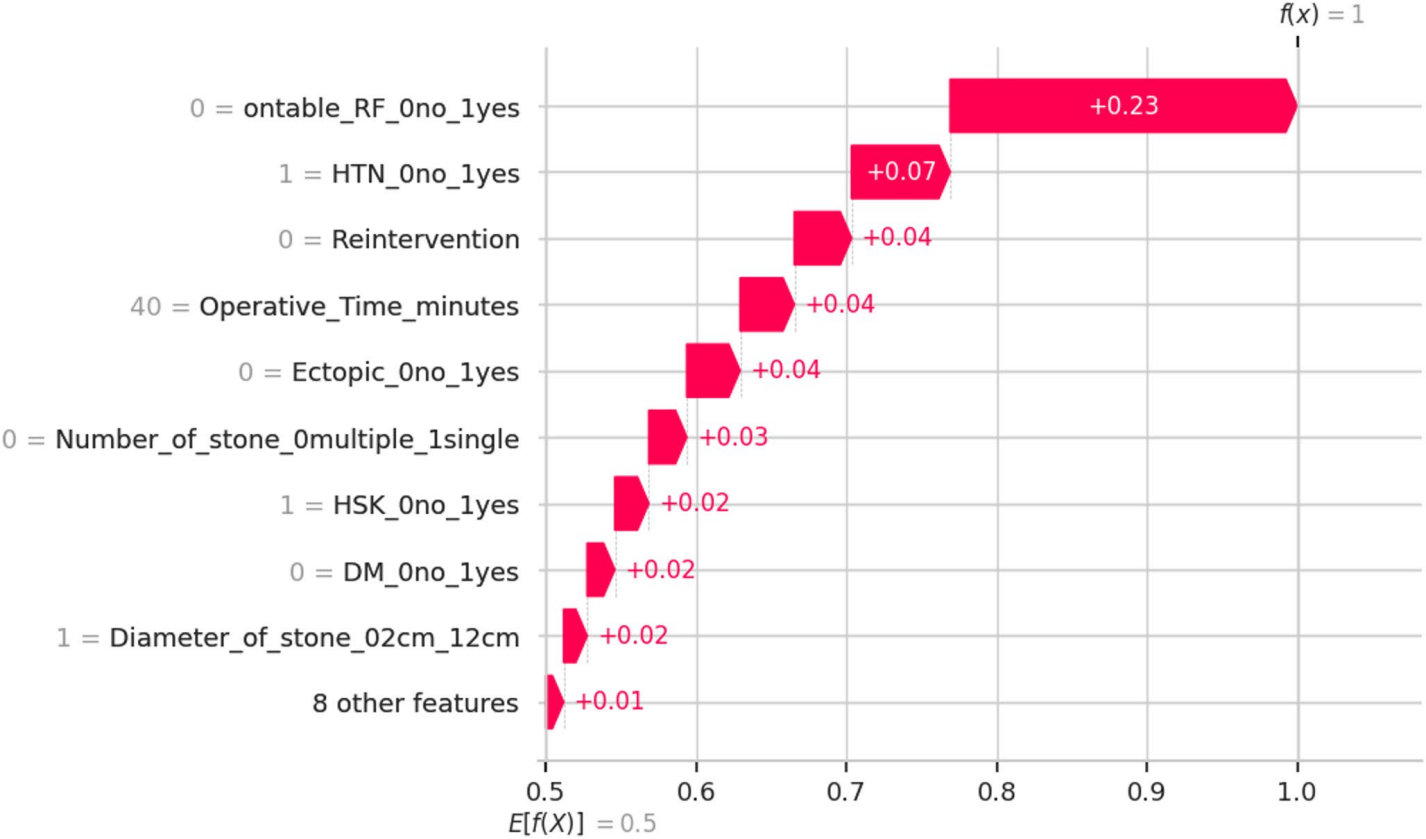
XAI: SHAP waterfall plot for SFS

The SHAP waterfall plot (Fig. 2) revealed how different features contributed to the final model prediction. The base value, representing the expected outcome before considering feature contributions, was 0.5. The most influential feature was on-table RF, which increased the model’s prediction by + 0.23. Other features, including operative time and need for reintervention, contributed smaller positive and negative values to the prediction. The final model output, after considering all feature contributions, was 1.0, reflecting the overall classification. This plot highlighted the cumulative effect of various features in driving the model’s prediction towards the final classification.

**Fig. 3:**
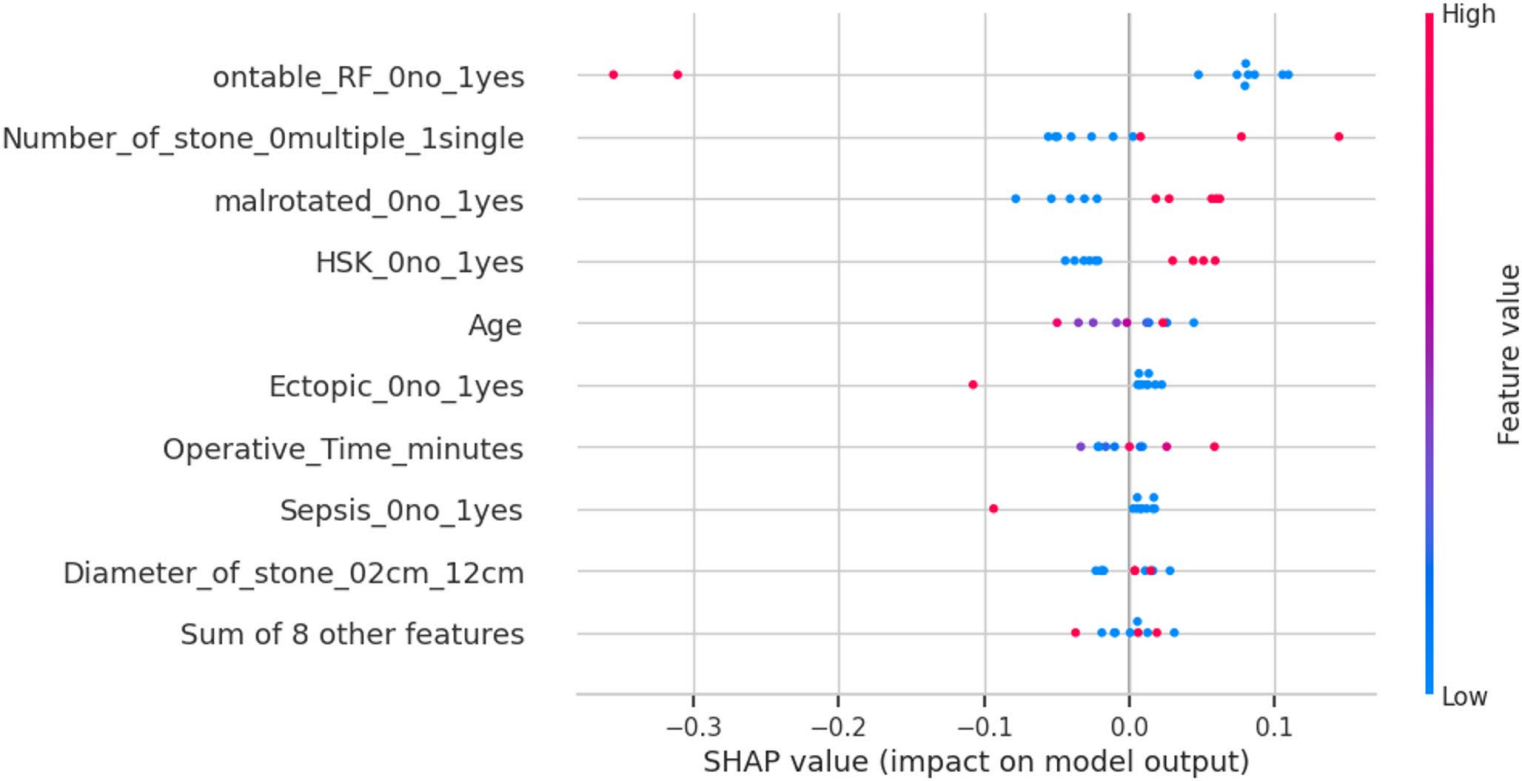
XAI: SHAP summary plot for SFS

The SHAP summary plot further emphasized the impact of different features on the model’s predictions. The plot showed that features such as on-table RF and the number of stones had a wide spread of SHAP values, indicating that their impact varied significantly depending on the instance. For instance, on-table RF exhibited both positive and negative contributions, suggesting its influence was context-dependent. Conversely, features like operative time and sepsis primarily contributed negative SHAP values, indicating that they tended to reduce the model’s output. This summary plot provided a comprehensive visualization of how each feature, and its value, contributed to the final prediction, offering valuable insights into the model’s decision-making process (see Fig. 3).

### Haematuria limiting vision

#### ML training and testing results

Top-performing models for haematuria prediction include XGBoost, Bagging Classifier, and CatBoost, with over 97% training accuracy and 93–94% validation accuracy. Decision Tree, Extra Trees, and Random Forest showed high accuracy but slight overfitting. Gradient Boosting and AdaBoost performed well with validation accuracy between 83 and 90%. SVM-Linear and Logistic Regression showed strong generalization with 76–78% validation accuracy. Overall, ensemble methods outperformed individual models, while linear models demonstrated solid generalization.

#### Analysis of correlation and logistic regression

The analysis of factors associated with haematuria after ureteroscopy in anomalous kidneys revealed several key correlations. Abandoned operations (*r* = 0.2353), reintervention (*r* = 0.2128), and on-table RF (*r* = 0.1718) were moderately linked to increased bleeding risk, highlighting the role of procedural complexity. Sepsis (*r* = 0.1211) also showed a mild positive correlation. Stone characteristics, including Hounsfield unit (HU) and diameter, had weak associations, while MK strongly reduced haematuria risk (*r*=-0.8352). Patient factors like age, sex, diabetes mellitus (DM), and operative time showed minimal impact. Overall, procedural complexity and anatomical features were more influential than patient characteristics in predicting haematuria.

The logistic regression model for haematuria had a Log-Likelihood of -95.220 and a low Pseudo R-squared of 0.0612, indicating weak predictive power. The model’s overall significance was not supported by the LLR p-value of 0.2583. hypertension (HTN) (coef = 0.9936, p = 0.067) showed a potential positive correlation with haematuria, while DM (coef=-1.1036, p = 0.171) suggested a negative association, though neither were statistically significant. “Sex” (coef=-0.5030, p = 0.130) also lacked significance. Issues with multicollinearity or missing data led to ‘nan’ values for some predictors. High standard errors further indicated instability in coefficient estimates.

### Postoperative sepsis

#### ML training and testing results

XGBoost led with 97.91% training accuracy, 93.86% validation accuracy, and a 71.44% F1-score. Gradient Boosting followed with 95.34% training and 92.10% validation accuracy, and a 67.88% F1-score, showing strong generalization. Decision Tree, Random Forest, and Extra Trees had high training accuracy (~ 98.16%) and solid validation (87–90%), but showed overfitting. CatBoost performed well with 97.05% training accuracy and 89.47% validation accuracy. Bagging Classifier had decent performance (97.42% training, 87.71% validation) but a lower F1-score (52.94%). AdaBoost offered good balance with 86.15% training and 83.33% validation accuracy, and a 54.06% F1-score. SVM-Linear, Logistic Regression, and LDA showed moderate training accuracy (~ 81–83%) but stable validation (~ 75–82%) and F1-scores (52–56%). Ensemble models, especially XGBoost, Gradient Boosting, and Random Forest, outperformed individual models, while linear models showed solid generalization. Tree-based models overfitted, and SVM-RBF, SVM-Poly, and Naïve Bayes consistently underperformed.

#### Analysis of correlation and logistic regression

The analysis of sepsis risk factors identified several moderate to weak correlations. Reintervention (*r* = 0.1723), longer operative times (*r* = 0.1702), and comorbidities like HTN (*r* = 0.1628) and DM (*r* = 0.1570) were moderate contributors, emphasizing the role of surgical complexity and patient conditions. Positive correlations were also found with on-table RF (*r* = 0.1257) and haematuria (*r* = 0.1211), suggesting they may increase infection risk. Conversely, factors like MK (*r*=-0.0216), larger stone diameter (*r*=-0.0980), more stones (*r*=-0.0504), and achieving stone-free status (*r*=-0.0501) showed weak negative correlations, hinting at a minor protective effect. Overall, procedural complexity and comorbidities were more influential in predicting sepsis than anatomical factors or stone characteristics.

The model performance metrics indicate a Log-Likelihood of -117.47, reflecting the model’s fit, with a higher (less negative) value being preferred. The Pseudo R-squared value of 0.1271 suggests weak predictive power, as it explains only a small portion of the variance in the data. However, the LLR p-value of 0.0001697 is highly significant, indicating that at least one predictor in the model is statistically significant.

### Abandoned operation

#### ML training and testing results

CatBoost was the top performer with 98.64% training and 98.25% validation accuracy, but its F1-score (49.56%) indicates class imbalance. XGBoost followed closely with similar accuracy (98.97% training, 97.36% validation) and an F1-score of 49.33%. Random Forest, Decision Tree, and Extra Trees had high accuracy (99.32% training, 97.36% validation) but showed signs of overfitting. Bagging Classifier performed well (98.75% training, 96.49% validation) with less overfitting. Gradient Boosting also performed solidly (97.85% training, 95.61% validation, 48.87% F1-score). Logistic Regression and LDA showed balanced performance with ~ 84% training and 85% validation accuracy, and F1-scores around 46–47%. AdaBoost had good generalization (96.26% training, 95.61% validation, 48.88% F1-score). CatBoost and XGBoost achieved best results, with Gradient Boosting, Bagging, and AdaBoost also performing well. Logistic Regression and LDA offered good generalization, while SVM-RBF and Naïve Bayes underperformed.

#### Analysis of correlation and logistic regression

HTN emerged as the strongest predictor of an abandoned operation (*r* = 0.6615), indicating a high risk in these patients. Other contributing factors included haematuria (*r* = 0.2353), on-table RF (*r* = 0.2105), and reintervention (*r* = 0.1898), reflecting the impact of procedural complexity. Operative time, DM, and sepsis showed weaker associations. In contrast, higher HU values, multiple stones, and achieving SFS were weakly protective. These results suggest that patient comorbidities and intraoperative challenges are key drivers of surgical abandonment.

The model performance metrics show a Log-Likelihood of -42.737, reflecting the model’s fit, with a higher (less negative) value indicating a better fit. The Pseudo R-squared value of 0.1913 indicates moderate predictive power, explaining 19.13% of the variance. The LLR p-value of 0.02724 suggests that at least one predictor is statistically significant. However, the warning about exceeding the maximum number of iterations indicates potential issues such as multicollinearity, imbalanced data, large feature coefficients, or poor scaling of numerical variables.

## Discussion

This is the first real-world evaluation of fURS outcomes in a cohort of patients affected by kidney anomalies, reporting findings of a ML predictive model. Our results offer valuable insights into the management of urolithiasis in patients with congenital renal anomalies using fURS. XAI tools, particularly decision trees and SHAP values, were instrumental in uncovering patterns that may otherwise be obscured by conventional statistical analysis.

Among the key takeaways, on-table RF emerged as the most impactful variable influencing model predictions, reflecting the importance in predicting medium and long term SFS, a result in line with current literature [6]. Operative time and patient’s age also showed strong contributions to the model’s output, with shorter operative durations and younger patients more frequently associated with anomalous kidneys, suggesting cautious procedural approaches or less complex stone burdens in those populations [7]. Moreover, a significant correlation between risk of incomplete clearance and stone burden, in line with previous studies, was found [8, 9]. Postoperative complications were also accurately predicted by our ML models. In particular, ML analysis achieved over 90% accuracy in predicting occurrence of sepsis, and a link was found with features such as operative time and concurrent comorbidities (HTN, DM).

One of the most striking observations is the relative ease with which kidney malrotation were managed compared to other anomaly types. The decision tree analysis indicated that MK cases were consistently classified with fewer branching decisions and lower Gini impurity, suggesting more predictable surgical outcomes. This is supported by clinical experience, as malrotation may affect orientation but often does not compromise ureteral access as severely as pelvic ectopic or horseshoe kidneys. In contrast, ectopic kidneys posed greater variability in classification and were associated with higher procedural complexity and heterogeneity in feature influence [10]. Horseshoe kidneys, while structurally distinct, exhibited moderate predictive clarity but still required nuanced consideration due to their variable vascular and ureteral anatomy [11, 12]. In this context, fURS has proven to be a safe and effective option to treat urolithiasis in MK, also when compared to PCNL [13].

The SHAP plots enriched this understanding by quantifying the individualized contribution of each feature to the model’s output. Features like the number of stones and the presence of an PEK demonstrated wide SHAP value distributions, indicating their context-specific impact on prediction. This reflects the real-world variability in anatomy and stone burden among patients with renal anomalies, emphasizing the need for personalized risk assessment.

### Limitations of the study

Despite these strengths, several limitations must be acknowledged. First, the retrospective design inherently limits causal inference and may be subject to selection and information bias. Second, the sample size, particularly for rare anomaly types like pelvic ectopic kidneys, may not be sufficient to generalize findings across broader populations. Nevertheless, being this the first report, and given the scarcity of literature evidence on the topic, we believe the sample to be considered adequate for the study. Third, the absence of standardized definitions for procedural success, complication grading, and anomaly classification across the literature hampers comparison and benchmarking. Additionally, while XAI improves transparency, it remains an evolving field, and interpretability does not replace the need for clinical validation. Future studies should also look at the cost and quality of life aspects of stone disease and AI integration into building predictive models.

Overall, this study highlights the utility of ML and XAI in enhancing our understanding of complex surgical populations. The insights provided by XAI can support more informed clinical decisions, especially in the absence of robust guidelines for URS in anatomically variant kidneys. Further prospective, multicentric studies are warranted to validate these findings and to guide the development of standardized treatment protocols for this unique patient cohort.

### Key findings

In this study, several key findings emerged regarding factors influencing SFS following fURS. Among anatomical variants, malrotated kidneys were associated with the most favourable outcomes, exhibiting more predictable and successful achievement of SFS compared to ectopic and horseshoe kidneys. The presence of RF at the end of surgery was identified as the strongest negative predictor of SFS, contributing approximately 30% to the model’s decision-making process and underscoring its clinical importance in evaluating procedural completeness. Higher SFS rates were also associated with shorter operative times and younger patient age, likely reflecting less complex stone burdens and reduced anatomical challenges. Conversely, ectopic and horseshoe kidneys were more frequently linked to lower SFS rates and increased uncertainty in predictions, highlighting the critical role of anatomical variation in procedural success. SHAP analysis revealed that specific features, such as the number of stones and type of anomaly, exerted patient-specific influences on outcomes, emphasizing the importance of individualized treatment planning. Furthermore, XAI methods enabled transparent and clinically interpretable predictions of SFS, providing an evidence-based framework to enhance preoperative risk stratification and guide patient counselling.

## Conclusion

This study used explainable AI to identify predictors SFS after fURS in patients with congenital renal anomalies. Malrotated kidneys had the highest SFS rates, suggesting they offer more favourable anatomy for stone clearance. In contrast, ectopic and horseshoe kidneys were linked to lower success and more residual fragments, the strongest negative predictor of SFS. Decision trees and SHAP plots provided transparent, clinically aligned insights into outcomes. These findings support the role of XAI in improving decision-making and highlight the need for prospective studies to standardize care in this complex population.

## Data Availability

No datasets were generated or analysed during the current study.
